# Effects of Combined Resistance and Aerobic Training on Arterial Stiffness in Postmenopausal Women: A Systematic Review

**DOI:** 10.3390/ijerph18189450

**Published:** 2021-09-07

**Authors:** Marko Manojlović, Branka Protić-Gava, Nebojša Maksimović, Tijana Šćepanović, Sunčica Poček, Roberto Roklicer, Patrik Drid

**Affiliations:** Faculty of Sport and Physical Education, University of Novi Sad, 21000 Novi Sad, Serbia; markomanojlovic1995@gmail.com (M.M.); brankapg@gmail.com (B.P.-G.); nebojsam@uns.ac.rs (N.M.); tijanascepanovic021@gmail.com (T.Š.); suncicapocekfsfv@gmail.com (S.P.); roklicer.r@gmail.com (R.R.)

**Keywords:** combined exercise, arterial stiffness, baPWV, postmenopausal women

## Abstract

The aim of this systematic review was to investigate the effects of combined resistance and aerobic exercise on arterial stiffness in postmenopausal women. Two databases, PubMed and Google Scholar were searched to identify relevant studies. The methodological quality was assessed with the Physiotherapy Evidence Database (PEDro) scale. Only seven studies met the eligibility criteria, and their outcomes were presented. Four studies demonstrated the effects of combined resistance and aerobic training, while three showed the effectiveness of exercise with both training components, aerobic and resistance. In all studies, arterial stiffness was measured by brachial–ankle pulse wave velocity (baPWV). Participants were middle-aged or older postmenopausal women of various health statuses (hypertensive, with comorbidities or healthy). The results unequivocally show that combined training reduces arterial stiffness. The most important finding of this review paper is that the applied type of exercise decreased baPWV in the range of 0.6–2.1 m/s. Moreover, combined resistance and aerobic exercise for 12 weeks, performed three times a week for about 60 min per training session, at a moderate intensity (40–60% HRR or HRmax), may be clinically meaningful to the cardiovascular system. In conclusion, we can say that combined resistance and aerobic training, or exercise with resistance and aerobic components, have important health implications for the prevention of cardiovascular disease and the maintenance or improvement of health in middle-aged and older postmenopausal women with different health conditions.

## 1. Introduction

Arterial stiffness depends on functional (endothelium, smooth muscle cells) and structural components (elastin, collagen, and connective tissue). Arterial stiffness is determined by structural and functional changes within the arterial walls, leading to increased pulse wave velocity (PWV) [1]. The measurement of PWV is globally accepted as the most simple, non-invasive, and reproducible method for arterial stiffness assessment [2]. There are several ways to quantify PWV. Carotid–femoral PWV (cfPWV) is a measure of arterial PWV through the whole aorta and the most recognized and established indicator of central arterial stiffness [1]. cfPWV is considered as the “gold standard”for arterial stiffness evaluation [2]. Brachial–ankle pulse wave velocity (baPWV), measured by analyses of the brachial and tibial arterial wave, is known as a unique measure of systematic arterial stiffness [3]. This method is easier to apply and requires less time than the gold standard for assessment arterial stiffness, cfPWV [4]. Mitchell et al. [5] showed that higher arterial stiffness is associated with increased risk for a first cardiovascular event. Increased baPWV by 1 m/s is related to a higher absolute risk of stroke by 0.9%, of death by 2.2%, and of coronary heart disease by 1.4% within 10–12 years [6]. Additionally, Lebrun et al. [6] noted an inverse relationship between high-density lipoproteins and arterial stiffness, while a positive correlation was observed among triglycerides, smoking, and baPWV. In healthy, postmenopausal women, increased arterial stiffness is significantly related to higher systolic and diastolic blood pressure [7]. On the other hand, postmenopausal women with osteoporosis may have elevated arterial stiffness and baPWV was negatively correlated with bone mineral density according to a study conducted by Sumino et al. [8]. Moreover, reduced muscle mass and increased visceral fat are associated with large artery stiffness in postmenopausal women with type 2 diabetes mellitus and in middle-aged adults [9,10]. Lower levels of arterial stiffness correlate with higher values of cardiorespiratory fitness and muscle strength not only in older adults but also in younger [11,12]. In general, increased arterial stiffness impairs the proper functioning of some body systems and disrupts the quality of life in women after menopause.

Physical activity (PA) is recommended and provides many benefits in postmenopausal women. Physical exercise has positive effects on bone health and is also an excellent strategy for the prevention and treatment of sarcopenia [13]. PA is negatively associated with arterial stiffness in postmenopausal women with normal weight, among middle-aged adults with hypertension, while sedentary time is positively associated with PWV in younger adults [14,15,16]. In addition, Sugawara et al. [17] suggest that both moderate and vigorous physical activities have favorable effects on central arterial stiffness in postmenopausal women. It is known that high-intensity resistance training elevates arterial stiffness in younger women [18], while moderate-intensity resistance training does not alter arterial stiffness in normotensive postmenopausal women [19]. However, aerobic training reduces arterial stiffness in postmenopausal women and female patients with metabolic syndrome [20,21]. Most importantly, combined aerobic and resistance training improves arterial compliance in postmenopausal women with type 2 diabetes mellitus [22]. An activity such as rowing, which contains elements of aerobic and resistance training, does not affect arterial stiffness in older men [23].

The purpose of this review paper is to synthesize studies that represent the effects of combined resistance and aerobic training or activities that have elements of aerobic and resistance exercise on arterial stiffness in postmenopausal women.

## 2. Materials and Methods

### 2.1. Search Strategy

The search process was conducted between March and May 2021. The following databases were searched: PubMed and Google Scholar. The search strategy was: “resistance training” OR “strength training” OR “aerobic training” OR “endurance exercise” OR “combined exercise” OR “aerobic-anaerobic activities” AND “arterial stiffness” OR “PWV” OR “pulse wave velocity” OR “baPWV” OR “brachial-ankle pulse wave velocity” OR “cfPWV” OR “carotid-femoral pulse wave velocity” OR “vascular stiffness” AND “menopause” OR “postmenopausal women” OR “middle-aged women” OR “older adults” OR “older women” OR “females”. Additionally, references of all selected articles and relevant systematic reviews were checked to identify other eligible studies. The guidelines of the PRISMA statement were followed.

### 2.2. Inclusion and Exclusion Criteria

Studies were appropriate for analysis if the following inclusion criteria were met: (a) studies published after 2010; (b) the experimental group performed a combined resistance and aerobic training, or that has aerobic–anaerobic components; (c) participants were only postmenopausal women; (d) arterial stiffness was measured by PWV (m/s); (e) control group had to be sedentary (no regular physical exercise); (f) studies written in English. Abstracts and meta-analyses were not considered. Studies were also excluded if: (a) they did not define a control group; (b) in addition to the training process, some nutritional supplements were consumed; (c) resistance and aerobic exercise were not performed on the same day.

### 2.3. Quality Assessment

The Physiotherapy Evidence Database (PEDro) scale was used to assess the methodological quality of the studies included in the review [24]. The PEDro scale has proven to be a valid measure of the methodological quality of clinical trials [25]. The PEDro scale consists of 10 different items such as random allocation, concealed allocation, baseline comparability, blinded subjects, blinded therapists, blinded assessors, adequate follow-up, intention-to-treat analysis, between-group comparison, point estimates, and variability. Given that subjects, therapists, or assessors can rarely be blinded, items 4–6 were removed from the scale. The maximum result of the modified PEDro scale is 7, and the lowest is 0.

### 2.4. Data Extraction

The following data were extracted from each study: first author, year of publication, sample size, characteristics of the study population (age and health status of postmenopausal women), description of the exercise program (training type, duration, frequency, intensity, volume), arterial stiffness measurement (PWV), exercise effects on artery stiffness, relationship between changes in arterial stiffness and blood pressure, and PEDro score.

### 2.5. Studies Selected

**Figure 1:**
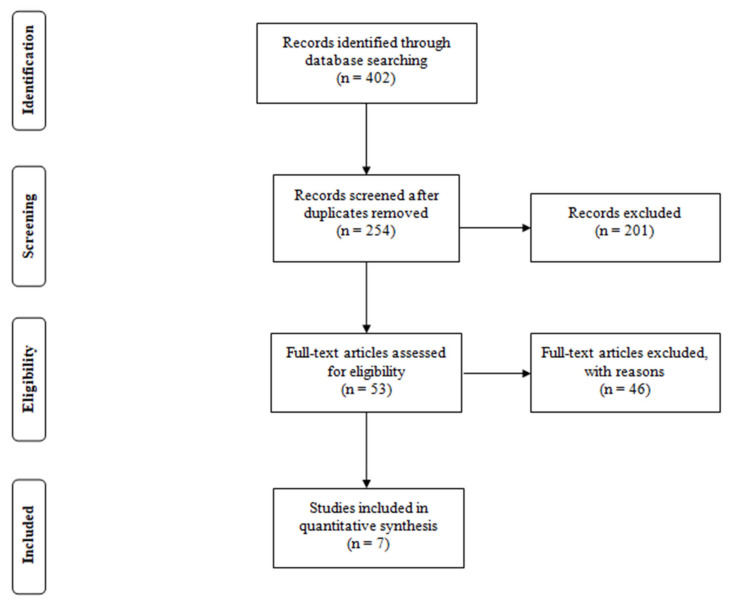
PRISMA flow diagram.

Overall, 402 studies were identified after searching the PubMed and Google Scholar databases. After duplicates were removed, 254 articles were screened, and 201 studies were excluded after reading abstracts and titles. The remaining 53 full-text articles were read and assessed for eligibility. Then, 46 studies were excluded for the following reasons: the training group did not apply combined resistance and aerobic exercise or aerobic–anaerobic activities; without a defined control group; arterial stiffness was measured by another method; the participants were premenopausal or women in the menstrual cycle; the experimental group consumed supplements along with exercise; articles were published before 2010. Finally, seven studies were included in the review paper (Figure 1).

### 2.6. Study Quality Assessment

The mean score on the PEDro scale was 5.3. Two articles met all items and had a score of 7. One study had a score of 6, and two studies did not fulfill two items: their score was 5. The remaining two articles had scores of 4 and 3. The overall results of the modified PEDro scale of all studies included in the review paper are presented in Table 1.

## 3. Results

**Figure 2:**
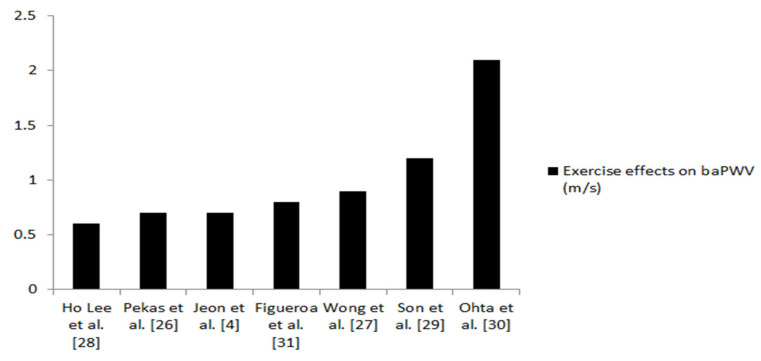
Effects of combined training on arterial stiffness (baPWV).

The main characteristics of included researches are presented in Table 2. All participants in the relevant articles were postmenopausal women. Four of them were hypertensive, at the first or second stage. In one included study, the sample consisted of women with comorbidities that consumed medications, while in the remaining two studies all were completely healthy. The sample size ranged from N = 16–41 postmenopausal women, while in one study there were 101 participants. All participants were middle-aged and older women. In all studies, arterial stiffness was measured by brachial–ankle pulse wave velocity (baPWV). Four articles showed the effects of combined resistance and aerobic exercise on baPWV [4,26,29,31], and three demonstrated the effectiveness of exercise that has a resistance component as well as aerobic [27,28,30]. The most important finding from this research is that the applied type of exercise reduced the level of arterial stiffness (baPWV) in the range of 0.6–2.1 m/s (Figure 2).

It is important to say that all studies analyzed the effects of combined exercise on blood pressure. Unfortunately, only two articles looked at associations between changes in arterial stiffness with changes in blood pressure. Wong et al. [27] showed that baPWV reduction was associated with a decrease only in systolic blood pressure (r = 0.66), but Jeon et al. [4] proved that changes in arterial stiffness were related to exercise effects on both systolic (r = 0.70) and diastolic (r = 0.65) blood pressure.

A detailed description of the exercises is visible in Table 3. Three articles demonstrated training with aerobic–anaerobic components (taekwondo, stair climbing, and bench step exercise); in the other four studies other combined resistance and aerobic training was performed. Jeon et al. [4] applied circuit resistance and aerobic training. All studies lasted 12 weeks, except the study conducted by Pekas et al. [26], which was conducted for 52 weeks. Participants usually practiced 3–4 times a week, for 40–60 min a day, except in the study done by Ohta et al. [30], where participants exercised 3 times a day, for 10–20 min. Exercise intensity was determined by % HRR, Ho Lee et al. [28]; Son et al. [29] demonstrated a gradual increase in intensity by weeks; age-predicted % HRmax; RPE Borg scale (6–20); % 1RM [31]; or with LA threshold [30].

## 4. Discussion

The purpose of this review study was to evaluate the effects of combined resistance and aerobic training, or exercise consisting of both aerobic and resistance components, on arterial stiffness in postmenopausal women. All seven articles unequivocally show that the mentioned exercise method reduces the level of arterial stiffness in postmenopausal women. In general, exercise for 12 weeks, applied three times a week, with lengths of one training session of about 60 min, at an intensity of 40–60% HRR or HRmax, decreased arterial stiffness measured by brachial–ankle pulse wave velocity (baPWV), in the magnitude of 0.6–2.1 m/s in middle-aged and older postmenopausal women with different health conditions. The most important finding of this review paper is that combined resistance and aerobic training, as well as exercise with resistance and aerobic components, reduced baPWV in the range of 0.6–2.1 m/s. Vlachopoulos et al. [32] demonstrated that a 1 m/s increase in baPWV was associated with a 14–15% increased risk of total CV events, CV mortality, and all-cause mortality, respectively. Higher baPWV was a predictive marker for CV events, especially ischemic stroke [33]. Additionally, PWV was an independent determinant of the longitudinal increase in systolic blood pressure [34]. Furthermore, elevated PWV has been associated with coronary, cerebral, and carotid atherosclerosis [35,36]. Therefore, reduced arterial stiffness measured over baPWV in the magnitude of 0.6–2.1 m/s after the implementation of combined resistance and aerobic exercise or training with resistance and aerobic components is considered clinically meaningful, primarily for the functioning of the cardiovascular system in healthy and hypertensive postmenopausal women. Otsuki et al. [37] showed that six weeks of combined aerobic and resistance exercise reduced baPWV in a sample of healthy older adults. Interestingly, aerobic training applied after high-intensity resistance exercise reduces PWV by 1 m/s [38] and neutralizes the effects of high-intensity resistance training, as previously shown [18]. Park et al. [39] used a similar training method: combined aerobic and resistance exercise that was realized in a period of 12 weeks, three times a week, at an intensity of 60–70% HRmax also decreased baPWV in older, obese men. Additionally, combined exercise reduced arterial stiffness in obese, prehypertensive, adolescent girls with similar values as in the presented study (baPWV −1.23 m/s) [40]. Rowing and taekwondo, exercises that include components of aerobic and resistance training, are also effective in decreasing arterial stiffness in middle-aged adults and obese adolescents [41,42]. Based on the presented information, it can be concluded that combined training of analog exercise intensity, as in our study, is equally effective in reducing PWV in populations close to postmenopausal women.

Swimming, performed by postmenopausal women with stage 2 hypertension, decreased carotid to radial PWV by 1.2 m/s [43]. The effects were in the same range as those for combined exercise. Ho et al. [44] demonstrated the efficiency of interval sprints in reducing arterial stiffness measured by baPWV and augmentation index (AIx) in overweight postmenopausal women. However, low-intensity resistance exercise did not alter arterial stiffness in obese postmenopausal women [45,46]. On the other hand, whole-body vibration training (WBVT), an alternative low-intensity resistance exercise, reduced baPWV in the range of 1.2–1.3 m/s and legPWV by 0.80 m/s in obese hypertensive and prehypertensive, or hypertensive postmenopausal women [47,48]. The decrease in legPWV was correlated with a reduction in ankle, brachial, and aortic systolic blood pressure [47]. It is obvious that different training methods, aerobic exercise, anaerobic or alternative low-intensity resistance exercise, similar to combined exercise, decrease arterial stiffness in postmenopausal women of diverse health status.

There are several possible mechanisms that could explain the reduction in arterial stiffness in postmenopausal women after combined resistance and aerobic training, and following other types of other exercise as well. Increased levels of circulating nitric oxide, a potent vasodilator, are evident after combined resistance and aerobic training in postmenopausal women [30,37]. In addition, aerobic exercise reduces asymmetric dimethylarginine, an inhibitor of nitric oxide synthase, leading to an increase in arterial compliance [49]. Matsubara et al. [21] showed that increased plasma Klotho gene concentration was associated with changes in the β-stiffness index in healthy postmenopausal women.

Finally, the limitations of this review paper should be considered. First, only two databases were searched, which may have limited the number of studies for inclusion. Second, in all studies, arterial stiffness was measured by baPWV, although cfPWV is the gold standard for assessing arterial stiffness. It is important to add that the previous report showed that baPWV has a significant positive correlation (r = 0.73) with cfPWV [50]. Third, on the assessment of methodological quality, a modified PEDro scale was used, and studies with a score < 4 were not excluded from the review. The strength of the research is that postmenopausal women had different health conditions, which indicates that the results can be generalized to a good number of postmenopausal women. The implications of the results are significant for middle-aged and older postmenopausal women.

## 5. Conclusions

The available evidence indicates that combined resistance and aerobic training, and activities with components of resistance and aerobic exercise, decrease arterial stiffness in postmenopausal women of different health statuses. The reduction is in the range of 0.6–2.1 m/s measured by baPWV, which may be clinically meaningful, probably reducing the risk of cardiovascular disease. Combined exercise in the already mentioned training design is recommended for maintaining and improving the health of middle-aged and older postmenopausal women. Future studies could investigate the effects of combined exercise on arterial stiffness in obese postmenopausal women.

## Figures and Tables

**Table 1:** Physiotherapy Evidence Database (PEDro) ratings of the included studies.

Study	1	2	3	4	5	6	7	8	9	10	Score
Pekas et al. [26]	−	+	−	x	x	x	+	+	+	+	5
Wong et al. [27]	+	+	+	x	x	x	−	−	+	+	5
Ho Lee et al. [28]	+	+	+	x	x	x	+	+	+	+	7
Jeon et al. [4]	−	−	+	x	x	x	−	−	+	+	3
Son et al. [29]	+	+	+	x	x	x	+	+	+	+	7
Ohta et al. [30]	+	−	+	x	x	x	−	−	+	+	4
Figueroa et al. [31]	+	−	+	x	x	x	+	+	+	+	6

Legend: 1—Random allocation; 2—Concealed allocation; 3—Baseline comparability; 4—Blind subjects; 5—Blind therapists; 6—Blind assessors; 7—Adequate follow-up; 8—Intention-to-treat analysis; 9—Between-group comparison; 10—Point estimates and variability. (+) items fulfilled; (-) items not fulfilled; (x) items removed.

**Table 2:** Characteristics of included studies.

Study	Participants	Age (Years)	Training Type	Outcomes	Training Effects on PWV
Pekas et al. [26]	Postmenopausal women with comorbidities and medication consumption, N = 101	77 ± 2	Combined resistance and aerobic exercise	baPWV	Decreased by 0.7 m/s
Wong et al. [27]	Postmenopausal women with stage 2 hypertension, N = 41	59 ± 1	Stair climbing training program	baPWV	Decreased by 0.9 m/s
Ho Lee et al. [28]	Postmenopausal women with stage 2 hypertension, N = 20	70 ± 4	Taekwondo training	baPWV	Decreased by 0.6 m/s
Jeon et al. [4]	Hypertensive postmenopausal women, N = 16	59 ± 1	Combined circuit resistance and aerobic training	baPWV	Decreased by 0.7 m/s
Son et al. [29]	Postmenopausal women with stage 1 hypertension, N = 20	75 ± 2	Combined resistance and aerobic training	baPWV	Decreased by 1.2 m/s
Ohta et al. [30]	Healthy postmenopausal women, N = 26	72 ± 1	Bench step exercise	baPWV	Decreased by 2.1 m/s
Figueroa et al. [31]	Healthy postmenopausal women, N = 24	54 ± 1	Combined circuit resistance and endurance exercise	baPWV	Decreased by 0.8 m/s

**Table 3:** Exercise description.

Study	Training Description	Weeks	Days per Week	Length of One Exercise Episode	Intensity
Pekas et al. [26]	Combined resistance and aerobic exercise; resistance (push up, seated row, leg press calf rises and others); aerobic (walking, jogging, cycling)	52	3	60 min	3 sets, 10–15 reps, RPE 12–14 for resistance training; 50–60% HRR, RPE 12–14 for aerobic exercise
Wong et al. [27]	Stair climbing program: climbing 192 steps 2 to 5 times/day (increased climbing every three weeks)	12	4	-	11–13 RPE on the 6–20 Borg scale
Ho Lee et al. [28]	Taekwondo training program: kicks, punches, steps, step sparring, taekwondo forms, walking, jogging, running	12	3	60 min	30–40% HRR for the first four weeks, 40–50% HRR for the second four weeks, 50–60% for the third four weeks
Jeon et al. [4]	Combined circuit resistance and aerobic training: 4 sets, 4 exercises: kettlebell exercise, squats and push- ups, core exercise, and step-box aerobic exercise	12	3	60 min	Mass of the kettlebell was 2 kg, aerobic exercise was performed at 65–80% of age-predicted maximal HR
Son et al. [29]	Combined resistance and aerobic training: various resistance band exercise (upper and lower), walking	12	3	60 min	Gradually increased from 40–50% HRR in 1–4 weeks to 60–70% HRR in 9–12 weeks
Ohta et al. [30]	Bench step exercise (home based): the height of the step bench was between 15 cm and 20 cm, and the step rhythm was initially set at 40 steps/min	12	3 times daily	10–20 min daily, 140 min/week	Level of LA and RPE: 6–20 Borg scale, end of exercise-LA > 4 mmol/L and/or RPE > 17
Figueroa et al. [31]	Combined circuit resistance and endurance exercise: one set of 12 repetitions for nine exercises on weight machines, and treadmill walking	12	3	40 min	60% of 1RM for resistance, and 60% of predicted HR maximum for aerobic component

Legend: HR-heart rate; HRR-heart rate reserve; RPE-rating of perceived exertion; LA-lactate threshold; RM-repetition maximum.
